# The Joining Behavior of Titanium and Q235 Steel Joined by Cold Metal Transfer Joining Technology

**DOI:** 10.3390/ma12152413

**Published:** 2019-07-29

**Authors:** Jinghuan Chang, Rui Cao, Yingjie Yan

**Affiliations:** State Key Laboratory of Advanced Processing and Recycling of Non-ferrous Metal, Lanzhou University of Technology, Lanzhou 730050, China

**Keywords:** welding-brazing, joining mechanism, strength, dissimilar metal

## Abstract

Cold metal transfer process is applied to join titanium and Q235 steel with copper filler metal. Scanning electron microscope (SEM), energy dispersive spectrometer (EDS) analysis, micro-hardness tests, and tensile strength test were performed to investigate the joining mechanism and strength of joints. The results show that the stacking order of two base metals affected the joining modes and strength. For top Q235 steel to bottom Ti-TA2 lapped joint, there was no distinct interface reaction layer between the steel base metal and the weld metal; dispersed TiFe_2_ intermetalics (IMCs) IMCs between the steel base metal and the Ti base metal greatly improved the strength of joint; the tensile force of the joint could reach up to 93% that of steel-steel joint using the same welding parameters. Additionally, the joints were fractured in dimple mode at the steel base metal. For top Ti-TA2 to bottom Q235 steel lapped joint, the increasing volume fraction of Ti-Cu IMCs at the Ti-Cu weld metal interface contributed to the strength of joint degradation. The joints under tensile loading are initiated at the Ti-Cu weld metal interface between the weld metal and Ti base metal, then propagated to weld metal, finally fractured with brittle mode.

## 1. Introduction

Titanium and titanium alloy, with their excellent corrosion resistance and high specific strength compared to steel, are widely applied in chemical and aerospace industries [1,2,3,4]. Zn coated steel is used due to their good corrosion resistance. The joints formed between titanium and steel have been widely used in chemical and nuclear industries, and both titanium and steel are greatly taken in manufacturing [5,6,7,8]. Therefore, joining titanium and steel is of great concern. However, it is difficult to successfully join titanium and steel directly based on the following two aspects: On the one hand, brittle intermetalics (IMCs) (TiFe and TiFe_2_) are formed due to low solubility of Fe in Ti (0.1 at. %, at room temperature) [1]. On the other hand, a significant mismatch of thermal expansion coefficients between these two materials results in large residual stress especially in the case of arc welding process with large heat input [9].

To obtain the Ti-steel joints, solid state joining methods such as diffusion bonding and friction welding were introduced. Friction welding process was used to inhibit the IMCs formed at the interface [5,10,11]. Diffusion bonding process was applied for the decrease in heterogeneities of chemical composition [12,13,14]. Although these solid-state joining methods were used to join titanium and steel, brittle Ti-Fe IMCs formed at interface are still unavoidable, which is compromised for the strength of Ti-steel joints. To further improve the mechanical properties of Ti-steel joints, various interlayers (Niobium, Tantalum, Silver, Nickel, and Vanadium) were applied for filler metal to reduce or even to prevent the formation of hard and brittle Ti-Fe IMCs [15,16,17,18,19,20]. Cherepanov et al. [15] investigated the strength of titanium-stainless steel joints formed by explosive welding with Nb and Ta foils as transition layers. It is shown that the highest ultimate tensile strength of 476 MPa and yield strength of 302 MPa were obtained with niobium foils. Lee [16] obtained the titanium-stainless steel joint with Ag-Cu alloy filler metal and an Ag interlayer. The results indicated that Ti base metal/TiAg/Ag-rich solid solution/steel base metal layered structure was formed in the welding joint. The joint was fractured along the layer with the ductile mode, and the strength was up to 440 MPa. Wang et al. [17] compared the microstructure and strength of electron beam welded titanium-steel joints with different filler metals (vanadium, nickel, copper, and silver), the results indicated that no Ti-Fe IMCs were formed at interface in the joints with nickel, silver, and copper filler metals. Additionally, the highest tensile strength of 310 MPa was obtained in the joint with silver filler metal. Reichardt et al. [18] used the laser deposition additive manufacturing method to fabricate Ti-6Al-4V-stainless steel gradient components with a vanadium interlayer.

Recently, ‘cold metal transfer’ (CMT) joining technique has been successfully applied to join dissimilar metals [21,22,23,24,25]. The key feature of the process is that the wire motion has been integrated into the joining process and into the overall control of the process. As a result, the lower heat input can be controlled, and thus the IMC formation and its thickness can be reduced as well, thereby enabling optimization of the joint strength. In the present study, due to low heat input of the key feature of CMT, CMT process was used to join pure titanium TA2 and hot dipped galvanized mild Q235 steel with ERCuNiAl copper-based wire as a filler metal to decrease the brittle Fe-Ti IMCs. Then the effects of the lapped sequence on joining mechanism and mechanical properties were investigated. To compare the weld appearance and mechanical properties, steel-steel lapped jointand Ti-Ti lapped joint with ERCuNiAl copper-based wire were also performed at the same welding parameters. In addition, reaction compounds, micro-hardness distribution, and fracture morphology of joints were investigated as well.

## 2. Experimental Procedure

### 2.1. Materials

Pure titanium TA2 and hot dipped galvanized mild Q235 steel with a thickness of 1mm were used in this study. The coating of hot galvanized steel was mainly composed of Zn solid solutions; Zn with a thickness of approximately 10 μm is the major element in the coating layer [25]. ERCuNiAl copper-based wire with a diameter of 1.2 mm was selected as the filler metal in this study.

Table 1 presents the physical properties and tensile strength of TA2 sheet and Q235 steel. Table 2 presents the nominal chemical composition of TA2 sheet, hot dipped galvanized mild Q235 steel, and ERCuNiAl copper-based wire. For the purpose of avoiding the influence of the surface contamination on the weldability, prior to welding, the hot dipped galvanized mild Q235 steel sheet was only degreased by acetone and alcohol. However, to avoid porosity produced in the welding processes, the pure titanium TA2 sheet was degreased by acetone first, and then polished by abrasive cloth followed by cleaning with 5–10% NaOH solution at a temperature range of 40~70 °C for 3–7 min and rinsed with tap water. This was followed by surface cleaning with 30% HNO_3_ solution at a temperature of 60 °C for 1–3 min, then rinsing with tap water and eventually rinsing with alcohol.

### 2.2. Welding Procedures

**Figure 1:**
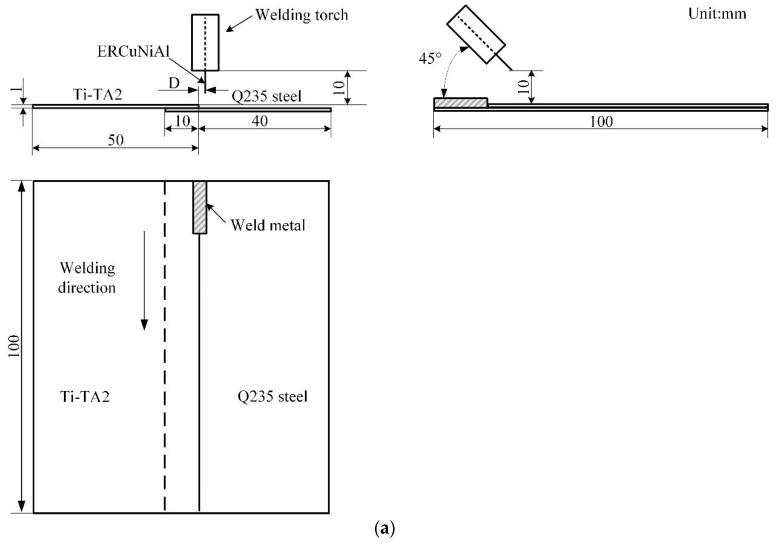

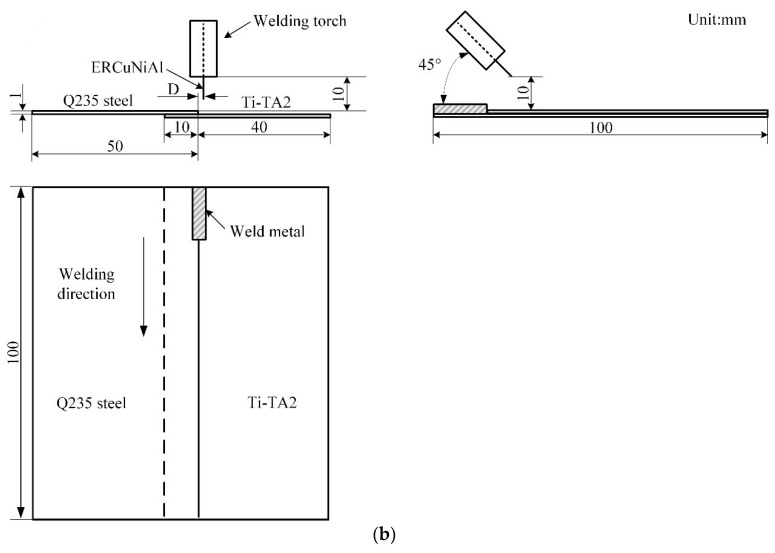
Schematic diagrams of joints (**a**) top Ti sheet-bottom steel sheet lapped joint (joint I) and (**b**) top steel sheet-bottom Ti sheet (joint II).

The lapped joint configurations, referred to in Figure 1, were fabricated from 100 mm × 50 mm sheets. As shown in Figure 1, configuration of top Ti-TA2 to bottom Q235 steel lapped joint (Joint I) (Figure 1a) and top Q235 steel to bottom Ti-TA2 lapped joint (Joint II) (Figure 1b) were adopted in the experiment with an overlap distance of 10 mm. The arrangement of the test sheets with respect to the weld torch was shown in Figure 1. The angle between the welding torch and the lap seam was 45° away from the direction of welding. The welding direction was parallel to the lap seam and was offset from the edge of the steel sheet edge by a deviation distance (D). All joints were welded using a Fronius arc welding system (CMT 3200) (Fronius, Austria). To obtain sound weld appearance and satisfied mechanical properties, various welding variables were used, as shown in Table 3. A 100% argon shielding gas with a flow rate of 15 L/min was used throughout the experiments. To compare the weld appearance and mechanical properties, steel-steel lapped joint (Joint III) and Ti-Ti joint (Joint IV) with Cu wire were also performed at the same welding parameters. The base metals of steel-steel lapped joint and Ti-Ti joint were hot dipped galvanized mild Q235 steel and TA2, respectively.

### 2.3. Analysis of Joining Mechanism

**Figure 2:**
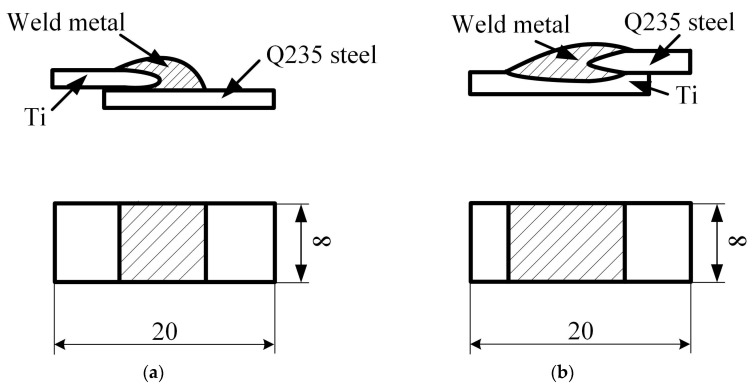
Dimensions (in mm) and configuration of cross-sections of the specimens: (**a**) Joint I (sample #1) and (**b**) Joint II (sample #2).

To examine the quality of CMT Ti-steel joints, the cross-sections of the specimens from the welded joints were prepared, the detailed dimensions and configuration of cross-sections of the specimens are shown in Figure 2. The microstructure and chemical component of the metallographic specimens were analyzed by Quanta FEG 450 (FEI Company, Hillsborough, OR, USA) scanning electron microscope (SEM) equipped with an energy dispersive spectrometer (EDS) system (Oxford Instrument, Oxford, UK). 

### 2.4. Mechanical Testing

**Figure 3:**
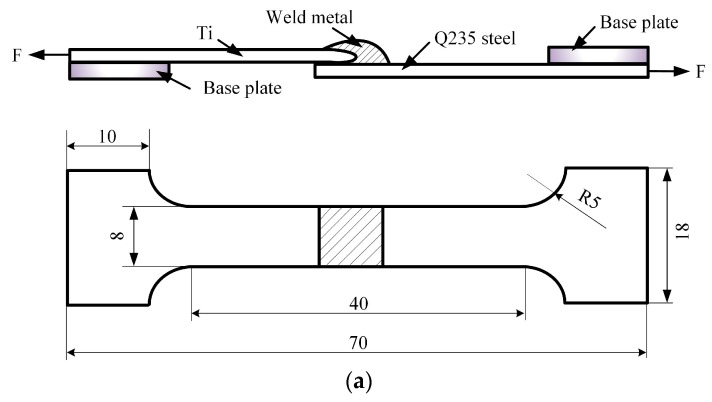

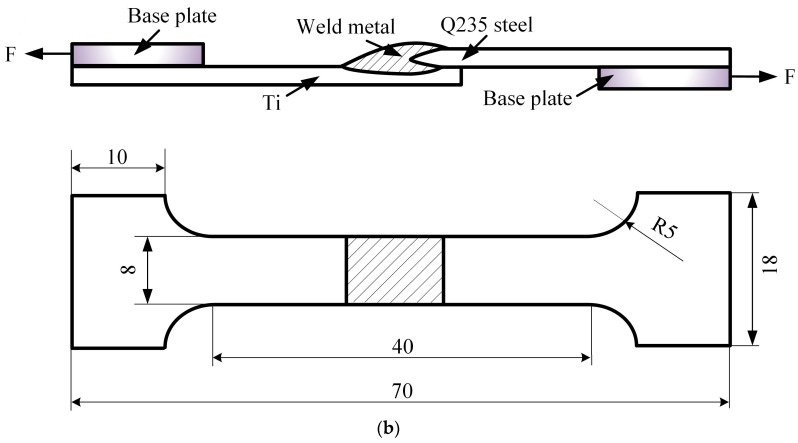
Dimensions (in mm) and configuration of the tensile samples: (**a**) Joint I (sample #1) and (**b**) Joint II (sample #2).

To investigate the mechanical properties of the welding joints, the tensile properties were evaluated by a testing machine with a speed of 0.5 mm∙min^−1^ at room temperature; specimens in Figure 3 were subjected to quasi-static tensile loading. To minimize bending stresses inherent in the testing, base plates were attached to both ends of the sample, as shown in Figure 3. For Joints I, II and III, three specimens were prepared for the testing experiment; the fracture morphology was observed by the SEM. The micro-hardness was measured by a HVT-1000A micro-hardness testing machine with the parameter HV 0.1/10 [26].

## 3. Results

### 3.1. The Weld Appearance of Joints

**Figure 4:**
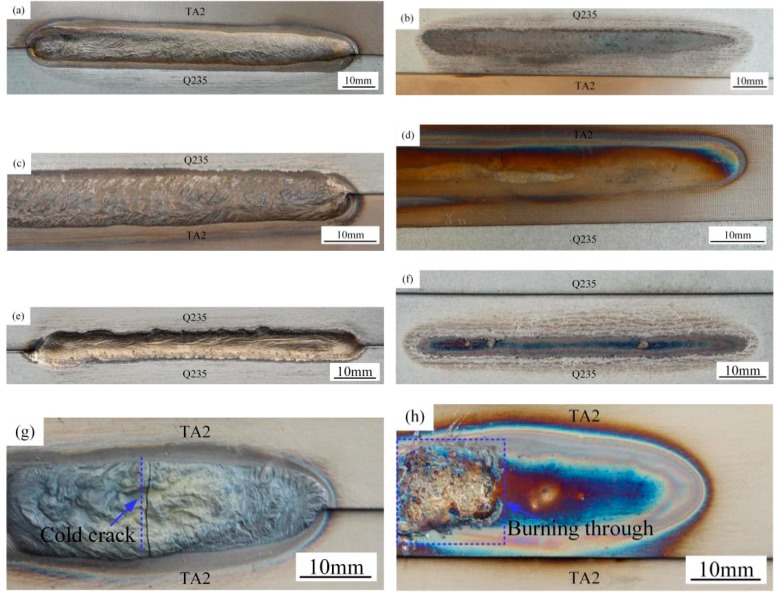
Weld appearance of (**a**) top view, (**b**) back view of joint I (sample #1), respectively; (**c**) top view, (**d**) back view of joint II (sample #2), respectively; (**e**) top view, (**f**) back view of joint III (sample #3), respectively; and (**g**) top view, (**h**) back view of joint IV (sample #4), respectively.

Figure 4 presents the weld appearance of top Ti-TA2 to bottom Q235 steel lapped joint (Joint I) and top Q235 steel to bottom Ti-TA2 lapped joint (Joint II). To compare the weld appearance and mechanical properties, steel-steel lapped joint and Ti-Ti lapped joint with Cu wire were also performed; the weld appearance of steel-steel lapped joint (Joint III) and Ti-Ti lapped joint (Joint IV) with Cu wire were also shown in Figure 4. Figure 4a,b presents the weld appearance of top view and back view of Joint I (sample #1) respectively. The weld with contiguous but no external cracks was obtained under desirable welding parameters (V_feed_ = 6 m∙min^−1^, V_welding_ = 8.53 mm∙s^−1^). No burning occurred in the back surface of the weld in Figure 4b. Figure 4c,d present the weld appearance of top view and back view of Joint II (sample #2) respectively. Continuous and uniform welds were formed with given welding parameters (V_feed_ = 6 m∙min^−1^, V_welding_ = 8.53 mm∙s^−1^). A slight burning occurred in the back surface of the weld in Figure 4d. Figure 4e,f shows the weld appearance of top view and back view of steel-steel lapped Joint III (sample #3) with Cu wire with same welding parameters (V_feed_ = 6 m∙min^−1^, V_welding_ = 8.53 mm∙s^−1^), respectively. Although the weld appearance of top view and back view were formed continuously, there was local undercut in the weld metal. Figure 4g,h presents the weld appearance of top view and back view of Ti-Ti lapped Joint IV (sample #4) with Cu wire under same welding parameters (V_feed_ = 6 m∙min^−1^, V_welding_ = 8.53 mm∙s^−1^). Cold crack and burning through were also produced in this joint. In comparison with the welds of Ti-steel and steel-steel, the wettability and spreadability of Ti-steel weld were better than that of steel-steel, as shown in Figure 4; both welds were well formed on the back, and there were no serious welding defects in the weld metal. However, the severe welding defects were produced in the Ti-Ti weld metal were due to poor heat conduction. In terms of four types of joints (Joints I, II, III, and IV), as shown in Figure 4, some welding defects were only formed in Joint IV. Therefore, the analyses focuse on the joining mechanism and strength of Joints I, II, and III in the following subsections.

### 3.2. Joining Mechanism of Joints

**Figure 5:**
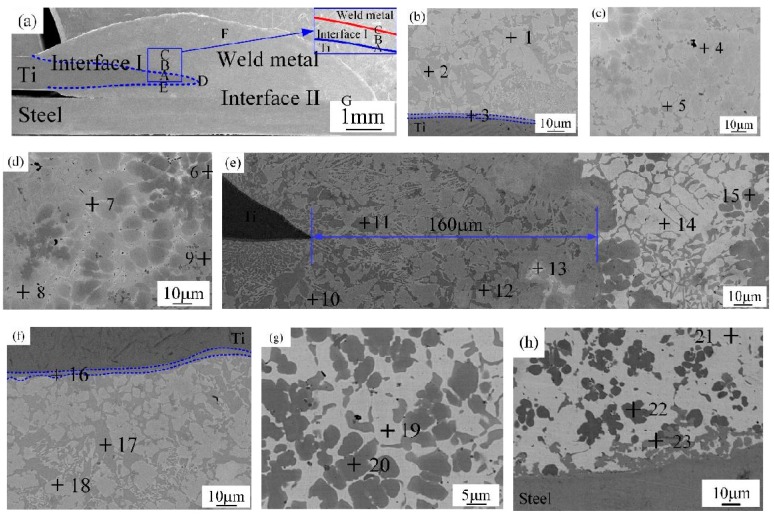
The microstructure of joint I (sample #1); (**a**) the cross section sample, (**b**) magnified zone A in Figure 5a, (**c**) magnified zone B in Figure 5a, (**d**) magnified zone C in Figure 5a, (**e**) magnified zone D in Figure 5a, (**f**) magnified zone E in Figure 5a, (**g**) magnified zone F in Figure 5a and (**h**) magnified zone G in Figure 5a.

**Figure 6:**
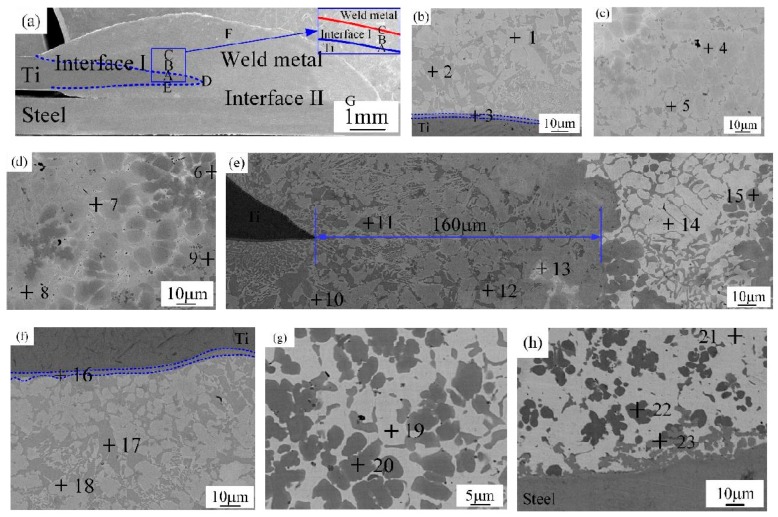
The microstructure of joint II (sample #2); (**a**) the cross-section sample, (**b**) magnified zone A in Figure 6a, (**c**) magnified zone B in Figure 6a, (**d**) magnified zone C in Figure 6a and (**e**) magnified zone D in Figure 6a.

**Figure 7:**
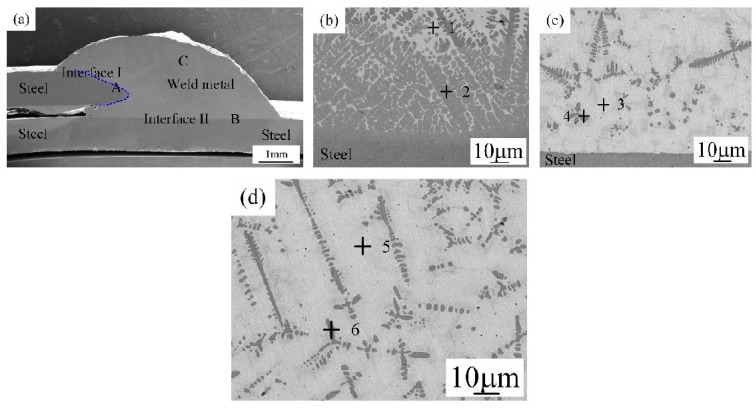
The microstructure of joint III (sample #3); (**a**) the cross-section sample, (**b**) magnified zone A in Figure 7a, (**c**) magnified zone B in Figure 7a and (**d**) magnified zone C in Figure 7a.

In order to study the joining mechanism of joints, a series of micrographs are shown in Figure 5, Figure 6 and Figure 7.

#### 3.2.1. Joint I (Top Ti-TA2 to Bottom Q235 Steel Lapped Joint)

The microstructure of Ti-steel joint (Joint I) is shown in Figure 5. There were five regions in the Joint I classified into steel base metal, Ti base metal, Interface I (Ti base metal-Cu weld metal), Interface II (Cu weld metal-steel base metal) and Cu weld metal, as shown in Figure 5a. To reveal the features of the Interface I, zones A–E in Figure 5a were systematically magnified in Figure 5b–f, respectively. Combined with the relevant phase diagrams [27,28], EDS was applied to analyze the components of various zones, and the results are presented in Table 4. In Figure 5b, zone A in Figure 5a is composed of light gray block CuTi_2_ and AlTi_3_ (marked by point 1), dark grey block CuTi_2_ IMCs phase (marked by point 2), and a continuous layer (marked by point 3) (blue dotted line in Figure 5b) is a dual-phased IMCs composed of CuTi_2_ and α-Ti solid solution. In Figure 5c, zone B in Figure 5a is composed of amounts of AlCu_2_Ti (marked by point 4) and dark gray CuTi_2_ (marked by point 5). In Figure 5d, zone C in Figure 5a is composed of Cu solid solution (marked by point 6), Ti_2_Cu_3_ and βTiCu_4_ (marked by point 7), Ti_2_Cu_3_ and Ti_3_Cu_4_ (marked by point 8), and AlCu_2_Ti (marked by point 9). In Figure 5e, zone D in Figure 5a is also a mixed zone with all kinds of phases, which consists of CuTi_2_ and AlTi_3_ (marked by point 10), CuTi_2_ (marked by point 11), AlCu_2_Ti (marked by point 12), Ti_2_Cu_3_ and βTiCu_4_ (marked by point 13), (Cu) and Al_3_Ti (marked by point 14), and Al-Ti-Cu-Fe-Ni phase (marked by point 15). In Figure 5f, zone E in Figure 5a is composed of CuTi_2_ and α-Ti (marked by point 16), CuTi_2_ (marked by point 17), and CuTi_2_ and AlTi_3_ (marked by point 18). Weld metal (zone F in Figure 5a) is magnified in Figure 5g, which is composed of dark grey Al-Ti-Cu-Fe-Ni phase (marked by point 20), and (Cu) and Al_3_Ti (marked by point 21). The Interface II (zone G in Figure 5a) is magnified in Figure 5h, which is composed of (Cu) and Al_3_Ti (marked by point 21), Al-Ti-Cu-Fe-Ni phase (marked by point 22) and Fe-Ti-Cu IMCs (marked by point 23). Phase analyses are in good agreement results referred in [21,29].

#### 3.2.2. Joint II (Top Q235 Steel to Bottom Ti-TA2 Lapped Joint)

The microstructure of steel-Ti joint (Joint II) is shown in Figure 6. Figure 6a presents the cross section of Joint II. From Figure 6a, the Joint II is composed of Interface I (zones A and B) between weld metal and steel sheet, Interface II (zone D) between weld metal and Ti sheet, and weld metal (zone C). The microstructures of various compositions are listed in Table 5. Zones A and B in the Interface I in Figure 6a are magnified in Figure 6b,c. As shown in Figure 6b, the zone A in the Interface I is composed of (Cu) and Al_3_Ti (marked by point 1) and Fe-Al-Cu IMCs (marked by point 2). In Figure 6c, the zone B in the Interface I is composed of TiFe_2_ IMCs phase (marked by point 3), (Cu) and Al_3_Ti (marked by point 4) and Fe-Al-Cu IMCs (marked by point 5), Weld metal zone C in Figure 6a is magnified in Figure 6d. From Figure 6d, the weld metal is composed of dark grey Al-Ti-Cu-Fe-Ni phase (marked by point 6) and (Cu) and Al_3_Ti (marked by point 7). The Interface II near to Ti sheet is magnified in Figure 6e, which is composed of (Ti) (HT) (marked by point 8), CuTi_2_ IMCs (marked by point 9), Fe-Ti-Cu IMCs (marked by point 10), and a continuous layer (blue dotted line in Figure 6e), which consists of CuTi_2_ and α-Ti (marked by point 11).

#### 3.2.3. Joint III (Q235 Steel to Q235 Steel Lapped Joint)

The microstructure of steel-steel joint (Joint III) is shown in Figure 7. Figure 7a presents the cross section of Joint III. From Figure 7a, the Joint III is composed of Interface I (zone A) between weld metal and top steel sheet, Interface II (zone B) between weld metal and bottom steel sheet, and weld metal (zone C). Table 6 presents the EDS analysis results of various zones in Figure 7a. Zones A and B in the Interface I and Interface II in Figure 7a were magnified in Figure 7b,c. The Interface I is magnified in Figure 7b, which is composed of (Cu) and Al_3_Ti (marked by point 1), and Fe-Al-Cu IMCs (marked by point 2). From Figure 7c, Interface II is composed of (Cu) and Al_3_Ti (marked by point 3), and Fe-Al-Cu IMCs (marked by point 4). The weld metal is composed of (Cu) and Al_3_Ti (marked by point 5), and Fe-Al-Cu IMCs (marked by point 6), as shown in Figure 7d.

### 3.3. Mechanical Properties of Joints

In order to analyze the effects of microstructure on properties, micro-hardness distribution along the given line in the cross section was examined, and tensile properties were evaluated by the tensile tests.

**Figure 8:**
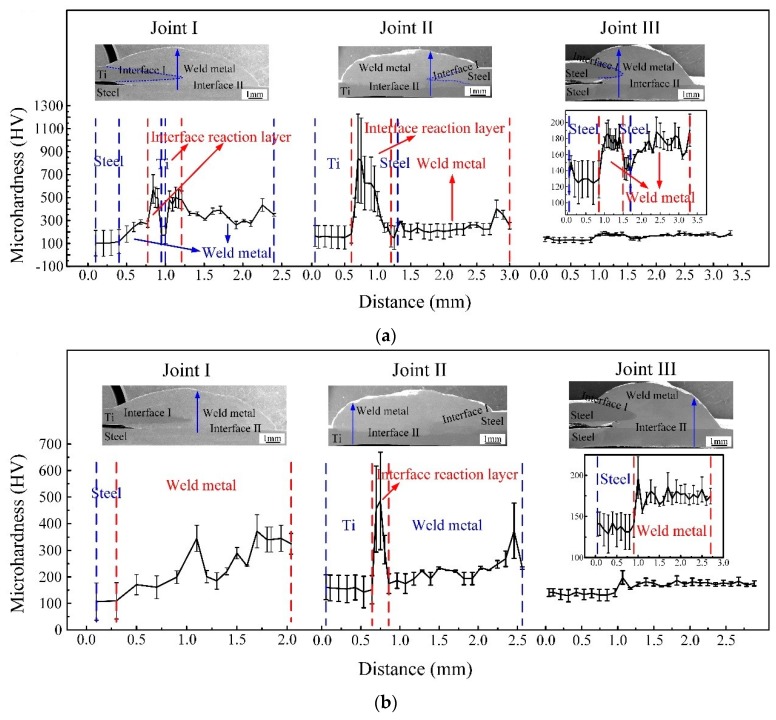
The micro-hardness variation diagram (**a**) along blue arrow in the cross section of Joint I (sample #1), joint II (sample #2), and joint III (sample #3), respectively and (**b**) from base metal to interface II to weld metal of Joint I (sample #1), joint II (sample #2), and joint III (sample #3), respectively.

Figure 8 is the micro-hardness value variation of joints. Figure 8a shows the micro-hardness variation along blue arrow in the cross section of Joints I, II and III, respectively. Regarding the Joint I, along with IMCs formed at the Ti-Cu weld metal interface, the micro-hardness of 500 HV in the interface reaction layer is higher than that of weld metal and base metal. Al-Ti-Cu-Fe-Ni phase and Cu solid solution were formed in the weld metal, which led to the low micro-hardness of weld metal. With regard to the Joint II, including Al-Ti-Cu-Fe-Ni phase and Cu solid solution, hard and brittle TiFe_2_ IMCs phase formed at Interface I (Ti-steel); the micro-hardness 800 HV in the interface reaction layer is higher than that of weld metal and base metal. As for the Joint III, there was no reaction layer at Interface I. The micro-hardness variation from base metal to Interface II to weld metal of Joints I, II, and III is shown in Figure 8b, respectively. In comparison with Joints I and III, there is no distinct interface reaction layer. And for Joint II, the micro-hardness of interface reaction layer was higher than that of weld metal and base metal because of Cu-Ti-Fe IMCs, CuTi_2_ IMCs, and Ti solid solution formed at Interface II.

**Figure 9:**
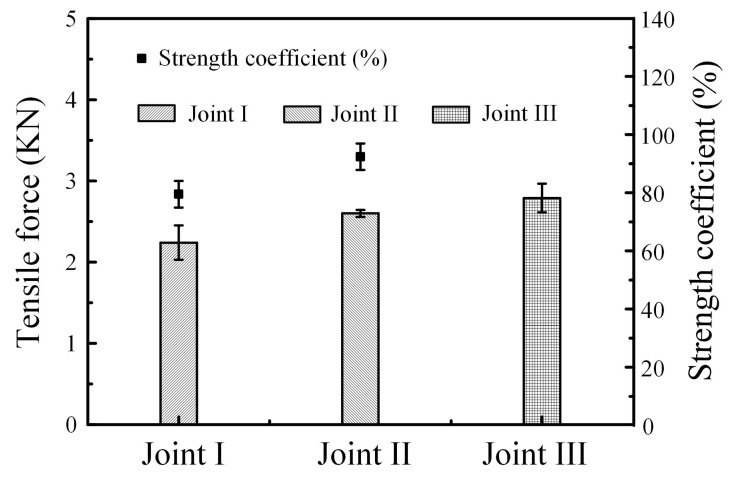
Tensile force in different joints listed in Table 3.

Figure 9 shows the tensile force of joints. In Figure 9, Joint I has low tensile force about 2.24 KN, but the tensile force of Joint II was greatly improved, and the value was 2.6 KN, which was close to tensile force of 2.79 KN for Joint III and reached 93% tensile force of steel-steel joint (Joint III) [30,31].

**Figure 10:**
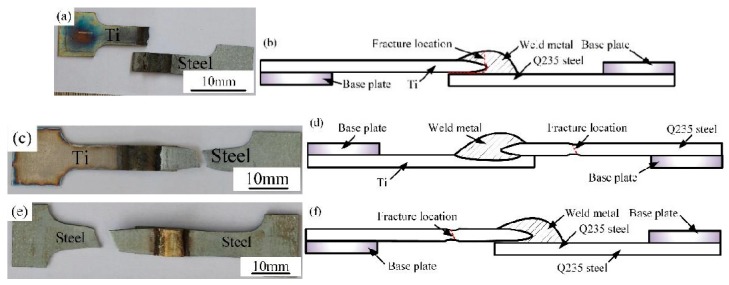
Fracture modes of the joints; (**a**–**b**) joint I (sample #1), (**c**–**d**) joint II (sample #2), (**e**–**f**) joint III (sample #3).

**Figure 11:**
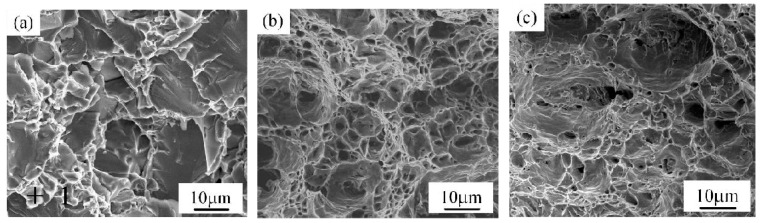
Fracture morphology of joints; (**a**) joint I (sample #1), (**b**) joint II (sample #2) and (**c**) joint III (sample #3).

**Figure 12:**
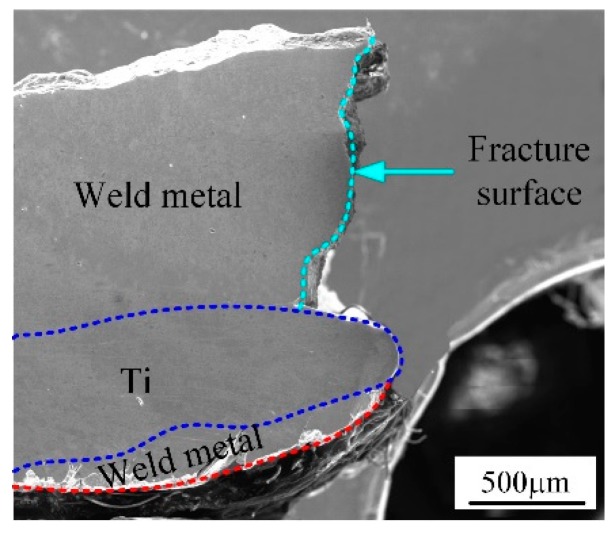
Fracture side of joint I.

In order to reveal the strengthening mechanism of Ti-steel Joints, fracture behaviors of different specimens were investigated as shown in Figure 10. To be noted, the corresponded fracture location is illustrated by a red line in Figure 10b,d and f. The Joint I was initiated at the Interface I between the weld metal and Ti base metal and propagated to weld metal. The Joint II was fractured at the steel base metal. The Joint III was also fractured at the steel base metal. In addition, the fracture surfaces of joints were displayed in Figure 11. In Figure 11a, the fracture of Joint I was primarily dominated with river patterns; correspondingly, the inclusions at the site of crack source were composed of AlCu_2_Ti, as shown in Table 7. The fracture of Joints II and III with a distinct dimple pattern were shown in Figure 11b,c. In Figure 12, it is obvious that the crack of Joints I was initiated at the Interface I (blue dotted line in Figure 5a) between the weld metal and Ti base metal, then propagated to weld metal, and finally fractured.

## 4. Discussion

As previously mentioned, there are significant differences on microstructure and properties between top Ti-TA2 to bottom Q235 steel lapped joint (Joint I) and top Q235 steel to bottom Ti-TA2 lapped joint (Joint II). To be specific, from Figure 5 and Figure 6 and Figure 8, Figure 9, Figure 10, Figure 11 and Figure 12, the effects of the stacking order of base metal on joining mechanism and mechanical properties were described as follows.

### 4.1. The Effect of the Stacking Order on the Joining Mechanism of the Ti-Steel Joints

Joint I and Joint II have the same connected interface I, II, and the weld metal, but the type of the compounds in the interface I for Joint I are significantly different from that of Joint II due to different lapped sequence. For Joint I as shown in Figure 5b–e, Interface I (Ti-Cu weld metal) was composed of five zones (zones A–E). A wide variety of IMCs were formed including phases of CuTi_2_, CuTi_2_ and AlTi_3_, AlCu_2_Ti, Ti_2_Cu_3_ and Ti_3_Cu_4_, Ti_2_Cu_3_ and βTiCu_4_, CuTi_2_ and α-Ti, and Cu solid solution. Many kinds of Ti-Cu IMCs formed in the Joint I lead to a significant increase in the micro-hardness, and the interface reaction layers display an average micro-hardness of 500 HV, as shown in Figure 8a,b. For Joint II, Interface I is composed of (Cu) and Al_3_Ti and Fe-Al-Cu IMCs, Al-Cu IMCs, (Cu) and Al_3_Ti, and TiFe_2_ IMCs, as shown in Figure 6b,c. For interface II, the compounds of two joints are remarkably different due to the different base metals on the bottom. The interface II of the Joint I is composed of Fe-Ti-Cu IMCs, but the interface II of the Joint II is made of Cu-Ti IMCs, causing the hardness of the interface II for Joint II higher than that of the interface II for Joint I.

### 4.2. The Effect of the Stacking Order on the Tensile Force of the Ti-Steel Joints

In Figure 9, it was found that the Joint II has higher tensile force than that of Joint I. For Joint I, an interface reaction layer with the thickness of 160 μm with CuTi_2_ and AlTi_3_, CuTi_2_, AlCu_2_Ti, Ti_2_Cu_3_ and βTiCu_4_, (Cu) and Al_3_Ti, and Al-Ti-Cu-Fe-Ni phase is formed in the direction parallel to the base metal, as shown in Figure 5e (zone D), as a result, the Joint I was initiated at the Interface I between the weld metal and Ti base metal, then propagated to weld metal, and finally fractured as shown in Figure 10b. A cleavage fracture was formed in the Ti–Cu IMC layer as shown in Figure 11a, which is consistent with Ref. [32,33]. Shen et al. [32] studied that the properties of TC4/oxygen-free copper with silver interlayer welded by diffusion bonding. The results presented that the weak component of the joints was formed at the Ag/OFC interface, then transferred into the Cu-Ti compound layers. Tashi et al. [33] reported vacuum brazing of Ti-6Al-4V and stainless steel using AgCuZn filler metal and found that the strength of joint was reduced due to the formation Ti-Cu and Fe-Cu-Ti IMCs. These studies revealed that Ti-Cu IMCs were detrimental to the strength of joints.

For joint II, although the micro-hardness value of interface reaction layer dispersed with TiFe_2_ IMCs reached 800 HV (Figure 8a), the tensile force of Joint II exhibited a high value of 2.6 KN. It was attributed to the dispersed TiFe_2_ IMCs strengthening the interface between the steel base metal and the Ti base metal [1]. Moreover, there was no distinct interface layer between the steel base metal and the weld metal, as shown in Figure 6b, and it was composed of (Cu) and Al_3_Ti, and Fe-Al-Cu IMCs. Finally, the Joint II is fractured at the steel base metal rather than the weld metal with minor IMCs and no obvious interface reaction layer.

## 5. Conclusions

CMT welding of 1 mm thick TA2 and 1 mm thick hot dipped galvanized mild Q235 steel with a diameter of 1.2 mm ERCuNiAl wire was carried out. The effects of the stacking order of base metal on the microstructure and mechanical properties of joints were investigated. Based on this study, the followed ideas can be drawn:

(1) A large number of Ti-Cu IMCs are formed at the Ti-Cu weld metal interface in top Ti-TA2 to bottom Q235 steel lapped joint, causing a significant increase in the micro-hardness of interface reaction layers than weld metal.
(2) The strength of the top Ti-TA2 to bottom Q235 steel lapped joint has the reduction due to the increasing volume fraction of Ti-Cu IMCs and an interface reaction layer with the thickness of 160 µm interface reaction layer. Additionally, the fracture is initiated at the Ti-Cu weld metal interface between the weld metal and Ti base metal, then propagated to weld metal, and finally fractured.
(3) The difference in microstructure of joints is responsible for the variation of tensile force. Maximum tensile force of 2.6 KN could be obtained in top Q235 steel to bottom Ti-TA2 lapped joint, which could reach up to 93% that of steel-steel joint. It was attributed to the dispersed TiFe_2_ IMCs strengthening the interface.
(4) The fracture of top Q235 steel to bottom Ti-TA2 lapped joint has a distinct dimple pattern. However, the fracture of top Ti-TA2 to bottom Q235 steel lapped joint exhibits a cleavage fracture mode containing AlCu_2_Ti IMCs.

## Figures and Tables

**Table 1:** Physical properties and tensile strength of Titanium and steel sheets.

Materials	Tm/°C	α/K^−1^	λ/W(m·K)^−1^	ρ/g∙cm^−3^	Crystal Lattice	σ_b_/MPa
TA2	1668	8.2 × 10^−6^	13.8	4.5	bcc/hcp	495
Q235	1535	11.5 × 10^−6^	66.7	7.86	bcc/fcc	235

**Table 2:** Nominal chemical compositions of TA2 sheet, hot dipped galvanized mild Q235 steel sheet and ERCuNiAl copper-based wire.

Materials	Element (wt. %)						
TA2	Fe	C	N	H	O	Ti	
	0.3	0.1	0.05	0.0015	0.25	Bal.	
Hot dipped galvanized mild Q235 steel	C	Si	Mn	P	S	Fe	Hot dipping Zn-coating (g∙m^−2^)
	0.01	0.01	0.39	0.30	0.025	Bal.	60
ERCuNiAl	Al	Ni	Fe	Pb	Mn	Cu	
	8.0	6.0	3.0	0.038	1.0	Bal.	

**Table 3:** CMT welding variables of TA2 and Q235 steel.

Sample No.	Wire-Feeder Speed (m∙min^−1^)	Weld Speed (mm∙s^−1^)	Heat Input (KJ∙mm^−1^)	Lap Jointing Type	Filler Metal
#1	6	8.53	0.170	type I	ERCuNiAl
#2	6	8.53	0.170	type II	ERCuNiAl
#3	6	8.53	0.170	–	ERCuNiAl
#4	6	8.53	0.170	–	ERCuNiAl

Note: type I—titanium sheet was placed on top of steel sheet, type II—steel sheet was placed on top of titanium sheet. The base metal of sample #3 was hot dipped galvanized mild Q235 steel, namely, the joint was steel-steel joint. The base metal of sample #4 was TA2, namely, the joint was Ti-Ti joint.

**Table 4:** EDS analysis results of various zones for joint I (specimen #1) in Figure 5 (at. %).

Point.	Al	Si	Ti	Fe	Cu	Zn	Ni	Possible Phase
1	6.68	0.34	47.15	8.58	34.55	1.24	3.47	CuTi_2_ and AlTi_3_
2	7.49	0.43	59.91	0.71	30.89	–	1.57	CuTi_2_
3	1.87	1.13	83.48	1.63	10.77	–	1.12	CuTi_2_ and α-Ti
4	21.50	0.21	26.09	5.24	45.44	0.88	0.65	AlCu_2_Ti
5	7.15	0.92	58.31	0.69	31.78	0.57	0.59	CuTi_2_
6	3.26	0.87	7.5	1.17	83.02	0.92	3.25	Cu solid solution
7	1.99	0.23	28.26	1.07	64.39	2.41	1.65	Ti_2_Cu_3_ and βTiCu_4_
8	1.28	0.67	37.16	2.82	53.08	1.53	3.46	Ti_2_Cu_3_ and Ti_3_Cu_4_
9	20.86	0.71	26.31	4.26	47.14	–	0.72	AlCu_2_Ti
10	7.84	–	44.54	7.58	34.49	1.21	4.35	CuTi_2_ and AlTi_3_
11	6.37	–	59.14	1.18	32.37	–	0.95	CuTi_2_
12	22.52	–	26.38	1.87	47.18	–	2.05	AlCu_2_Ti
13	1.76	–	33.62	1.12	62.25	–	1.25	Ti_2_Cu_3_ and βTiCu_4_
14	10.29	–	2.25	1.19	84.87	–	1.40	(Cu) and Al_3_Ti
15	22.91	–	24.85	17.70	22.48	0.95	11.12	Al-Ti-Cu-Fe-Ni phase
16	2.80	0.77	82.02	1.76	11.06	0.83	0.76	CuTi_2_ and α-Ti
17	8.61	–	56.06	1.51	29.87	1.53	2.42	CuTi_2_
18	7.82	–	45.23	8.27	34.09	0.88	3.70	CuTi_2_ and AlTi_3_
19	11.30	–	2.69	1.02	81.64	2.21	1.15	(Cu) and Al_3_Ti
20	24.95	–	23.84	18.25	22.19	0.58	10.19	Al-Ti-Cu-Fe-Ni phase
21	13.73	0.49	1.03	1.75	78.63	2.57	1.69	(Cu) and Al_3_Ti
22	23.86	0.42	23.39	20.40	17.49	0.68	13.75	Al-Ti-Cu-Fe-Ni phase
23	7.25	1.50	24.06	51.33	12.16	1.30	2.40	Fe-Ti-Cu IMCs

**Table 5:** EDS analysis results of various zones for joint II (specimen #2) in Figure 6 (at. %).

Point.	Al	Si	Ti	Fe	Cu	Zn	Ni	Possible Phase
1	17.36	1.27	1.29	6.37	70.53	0.55	2.62	(Cu) and Al_3_Ti
2	12.01	0.54	6.87	64.87	11.31	1.21	3.19	Fe-Al-Cu IMCs
3	2.35	–	30.64	62.46	2.24	2.30	–	TiFe_2_
4	15.34	–	1.75	4.50	75.77	–	2.65	(Cu) and Al_3_Ti
5	15.32	–	7.58	60.60	13.09	–	3.41	Fe-Al-Cu IMCs
6	28.88	–	21.65	20.24	14.23	–	15.00	Al-Ti-Cu-Fe-Ni phase
7	16.87	–	1.43	2.26	77.27	0.48	1.69	(Cu) and Al_3_Ti
8	11.21	0.88	62.92	4.61	16.88	1.41	2.10	(Ti)(HT)
9	7.14	0.77	56.79	1.71	29.38	1.72	2.49	CuTi_2_
10	7.64	1.78	51.44	11.77	24.50	0.52	2.35	Fe-Ti-Cu IMCs
11	2.10	1.64	80.45	1.24	11.97	–	1.43	CuTi_2_ and α-Ti

**Table 6:** EDS analysis results of various zones for joint III (specimen #3) in Figure 7 (at. %).

Point.	Al	Fe	Cu	Zn	Ni	Possible Phase
1	15.00	9.63	68.54	3.42	3.42	(Cu) and Al_3_Ti
2	8.18	76.60	11.55	1.71	1.95	Fe-Al-Cu IMCs
3	18.47	4.20	69.04	3.62	4.67	(Cu) and Al_3_Ti
4	18.07	59.73	13.43	2.59	6.19	Fe-Al-Cu IMCs
5	16.72	3.92	72.21	3.20	3.95	(Cu) and Al_3_Ti
6	19.35	60.74	15.38	–	4.53	Fe-Al-Cu IMCs

**Table 7:** EDS analysis results of fracture morphology for joint I (specimen #1) in Figure 11 (at. %).

Point.	Al	Si	Ti	Fe	Cu	Zn	Ni	Possible Phase
1	19.5	0.5	27.6	5.9	40.2	–	6.3	AlCu_2_Ti

## References

[1] Guo S., Zhou Q., Peng Y., Xu X., Diao C., Kong J., Luo T., Wang K., Zhu J. (2015). Study on strengthening mechanism of Ti/Cu electron beam welding. Mater. Des..

[2] Balasubramanian M. (2015). Application of Box–Behnken design for fabrication of titanium alloy and 304 stainless steel joints with silver interlayer by diffusion bonding. Mater. Des..

[3] Vigraman T., Ravindran D., Narayanasamy R. (2012). Effect of phase transformation and intermetallic compounds on the microstructure and tensile strength properties of diffusion-bonded joints between Ti–6Al–4V and AISI 304L. Mater. Des..

[4] Balasubramanian M., Jayabalan V., Balasubramanian V. (2008). Developing mathematical models to predict tensile properties of pulsed current gas tungsten arc welded Ti-6Al-4V alloy. Mater. Des..

[5] Li X., Li J., Liao Z., Jin F., Zhang F., Xiong J. (2016). Microstructure evolution and mechanical properties of rotary friction welded TC4/SUS321 joints at various rotation speeds. Mater. Des..

[6] Cao R., Huang Q., Chen J.H., Wang P.C. (2014). Cold metal transfer spot plug welding of AA6061-T6-to-galvanized steel for automotive applications. J. Alloys Compd..

[7] Liu J.G., Cai W.C., Liu L., Han J.T., Liu J. (2017). Investigation of interfacial structure and mechanical properties of titanium clad steel sheets prepared by a brazing-rolling process. Mater. Sci. Eng. A.

[8] Li C., Shi Y., Gu Y., Yuan P. (2018). Monitoring weld pool oscillation using reflected laser pattern in gas tungsten arc welding. Mater. Des..

[9] Liu K., Li Y., Wang J. (2016). Improving the Interfacial Microstructure Evolution of Ti/Stainless Steel GTA Welding Joint by Employing Cu Filler Metal. Mater. Manuf. Process..

[10] Li P., Li J., Salman M., Liang L., Xiong J., Zhang F. (2014). Effect of friction time on mechanical and metallurgical properties of continuous drive friction welded Ti6Al4V/SUS321 joints. Mater. Des..

[11] Akbarimousavi S.A.A., GohariKia M. (2011). Investigations on the mechanical properties and microstructure of dissimilar cp-titanium and AISI 316L austenitic stainless steel continuous friction welds. Mater. Des..

[12] Kundu S., Roy D., Chatterjee S., Olson D., Mishra B. (2012). Influence of interface microstructure on the mechanical properties of titanium/17-4 PH stainless steel solid state diffusion bonded joints. Mater. Des..

[13] Velmurugan C., Senthilkumar V., Sarala S., Arivarasan J. (2016). Low temperature diffusion bonding of Ti-6Al-4V and duplex stainless steel. J. Mater. Process. Technol..

[14] Kundu S., Mishra B., Olson D.L., Chatterjee S. (2013). Interfacial reactions and strength properties of diffusion bonded joints of Ti64 alloy and 17-4PH stainless steel using nickel alloy interlayer. Mater. Des..

[15] Cherepanov A.N., Mali V.I., Maliutina I.N., Orishich A.M., Malikov A.G., Drozdov V.O. (2016). Laser welding of stainless steel to titanium using explosively welded composite inserts. Int. J. Adv. Manuf. Technol..

[16] Lee J.G., Lee M.K. (2017). Microstructure and mechanical behavior of a titanium-to-stainless steel dissimilar joint brazed with Ag-Cu alloy filler and an Ag interlayer. Mater. Charact..

[17] Wang T., Zhang B., Wang H., Feng J. (2014). Microstructures and Mechanical Properties of Electron Beam-Welded Titanium-Steel Joints with Vanadium, Nickel, Copper and Silver Filler Metals. J. Mater. Eng. Perform..

[18] Reichardt A., Dillon R.P., Borgonia J.P., Shapiro A.A., McEnerney B.W., Momose T., Hosemann P. (2016). Development and characterization of Ti-6Al-4V to 304L stainless steel gradient components fabricated with laser deposition additive manufacturing. Mater. Des..

[19] Tomashchuk I., Grevey D., Sallamand P. (2015). Dissimilar laser welding of AISI 316L stainless steel to Ti6-Al4-6V alloy via pure vanadium interlayer. Mater. Sci. Eng. A.

[20] Jing Y., Yang Q., Xiong W., Huang B., Li B., Zhang M. (2016). Microstructure and shear strength of brazed joints between Ti(C, N)-based cermet and steel with Cu-Ag-Ti filler metal. J. Alloys Compd..

[21] Cao R., Feng Z., Chen J.H. (2014). Microstructures and properties of titanium–copper lap welded joints by cold metal transfer technology. Mater. Des..

[22] Cao R., Wen B.F., Chen J.H., Wang P.C. (2013). Cold Metal Transfer joining of magnesium AZ31B-to-aluminum A6061-T6. Mater. Sci. Eng. A.

[23] Cao R., Yu G., Chen J.H., Wang P.C. (2013). Cold metal transfer joining aluminum alloys-to-galvanized mild steel. J. Mater. Process. Technol..

[24] Cao R., Chang J.H., Zhu H.X., Mao G.J., Xu Q.W., Shi Y., Chen J.H., Wang P.C. (2018). Investigation of wire selection for CMT plug joining Mg AZ31-to-galvanized steel. J. Manuf. Process..

[25] Cao R., Chang J.H., Huang Q., Zhang X.B., Yan Y.J., Chen J.H. (2018). Behaviors and effects of Zn coating on welding-brazing process of Al-Steel and Mg-steel dissimilar metals. J. Manuf. Process..

[26] (2005). BS EN ISO 6507–1 (2005).

[27] Massalski T.B. (1990). Binary Alloy Phase Diagrams.

[28] Prince A., Villars P., Okamoto H. (1995). Handbook of Ternary Phase Alloys.

[29] Cao R., Feng Z., Lin Q., Chen J.H. (2014). Study on cold metal transfer welding–brazing of titanium to copper. Mater. Des..

[30] Liu Y., Sun Q., Liu J., Wang S., Feng J. (2015). Effect of axial external magnetic field on cold metal transfer welds of aluminum alloy and stainless steel. Mater. Lett..

[31] Chen R., Wang C., Jiang P., Shao X., Zhao Z., Gao Z., Yue C. (2016). Effect of axial magnetic field in the laser beam welding of stainless steel to aluminum alloy. Mater. Des..

[32] Shen Q., Xiang H., Luo G., Wang C., Li M., Zhang L. (2014). Microstructure and mechanical properties of TC4/oxygen-free copper joint with silver interlayer prepared by diffusion bonding. Mater. Sci. Eng. A.

[33] Tashi R.S., Mousavi S., Atabaki M.M. (2014). Diffusion brazing of Ti-6Al-4V and austenitic stainless steel using silver-based interlayer. Mater. Des..

